# Session-by-session change in misophonia: a descriptive case study using intensive CBT

**DOI:** 10.1017/S1754470X23000107

**Published:** 2023-07-21

**Authors:** Jane Gregory, Chloe Foster

**Affiliations:** 1 Department of Experimental Psychology, University of Oxford; 2 Oxford Health NHS Foundation Trust; 3 South London and Maudsley NHS Foundation Trust

**Keywords:** Case formulation, Case study, CBT, Measurement, Transdiagnostic

## Abstract

There is preliminary evidence that CBT may be helpful for improving symptoms of misophonia, but the key mechanisms of change are not yet known for this disorder of decreased tolerance to everyday sounds. This detailed case study aimed to describe the delivery of intensive, formulation-driven CBT for an individual with misophonia and report on session-by-session outcomes using a multidimensional measurement tool (S-Five). The patient was offered twelve hours of treatment over five sessions, using transdiagnostic and misophonia-specific interventions. Reliable and clinically significant change was found from baseline to one-month follow up. Visual inspection of outcome graphs indicated that change occurred on the “outbursts” and “internalising appraisals” S-Five subscales following assessment, and on the “emotional threat” subscale after first treatment session. The other two subscales started and remained below a clinically significant level. The biggest symptom change appeared to have occurred after second session, which included interventions engaging with trigger sounds. The results demonstrated the individualised nature of misophonia, supporting the use of individually tailored treatment for misophonia and highlighting the importance of using a multidimensional measurement tool.

## Introduction

Misophonia, a decreased tolerance to certain sounds, can cause significant distress and functional impairment (Jager, de Koning, et al., 2020a; Jastreboff & Jastreboff, 2002; Swedo et al., 2021). Common “triggers” include sounds that are often encountered on a daily basis, such as eating, breathing, tapping and rustling sounds (Hansen et al., 2021). The acoustic properties of sounds appear to be secondary to the meaning applied to sounds, with reactions potentially influenced by context, prior experience, personality factors, mood and energy levels, as well as predictions about the sound, the perpetrator and the impact of the sound on the individual (Jastreboff & Jastreboff, 2015).

There is preliminary evidence that cognitive behavioural therapy (CBT) may be helpful for reducing the distress and impairment associated with misophonia (Jager, Vulink, et al., 2020b). However, it is not yet known which specific interventions are most effective, nor do we understand the mechanisms of change for this disorder. A range of CBT strategies have been employed, including attention training (Bernstein et al., 2013; Schröder et al., 2017), counterconditioning, stimulus manipulation (Schröder et al., 2017), exercises involving exposure to sounds, cognitive restructuring (McGuire et al., 2015; Reid et al., 2016) and acceptance of reactions to sounds (Schneider & Arch, 2017).

Frank and McKay (2019) proposed employing inhibitory learning strategies for treatment of misophonia. They report a protocol for a pilot randomized trial that involves experiments intended to violate the patient’s expected outcome when they encounter aversive sounds, with the aim of creating new associations with these sounds to compete with existing associations. This includes using novelty and humour as part of experiments, as well as removing safety-seeking behaviours, modifying and combining stimuli and applying the exercises to different contexts.

Considering we do not yet understand the mechanisms maintaining disorder-level misophonia, a formulation-driven approach to treatment makes sense. Common practice in the UK, a formulation-driven approach to CBT involves the therapist and patient developing a shared understanding of the problem, identifying potential causal and maintenance factors and developing a treatment plan in line with this conceptualisation and the individual’s goals (Bieling & Kuyken, 2003). This approach enables us to use empirically grounded interventions from well-researched disorders and transdiagnostic processes, reviewing and refining the formulation (also known as case conceptualisation) and treatment plan in line with new evidence that emerges from therapy (Salkovskis, 2002). The patient, an expert in their experience of misophonia, would work together with the therapist to design experiments to test their shared theory of what maintains the problem and attempt to disrupt these cycles.

As yet, there is little information available about the characteristics of change in this patient group. In the case studies and clinical trials published to date, symptom change has been measured using tools that do not appear to capture the complexity of the disorder reported by patients. Recently published multidimensional psychometric tools for misophonia (Rosenthal et al., 2021; Vitoratou et al., 2021) show promise, although these have not been assessed for responsiveness or sensitivity to change, that is, the capacity to detect symptom change in patients and to measure the magnitude of that change (Kalsi-Ryan et al., 2016). This feature of psychometric tools is important for measuring effectiveness of intervention in randomised trials and in clinical practice and measuring change requires a good understanding of the change characteristics of patients in response to intervention (Stratford & Riddle, 2005). Individual case studies can support this process, by providing detailed descriptions of session-by-session change alongside scores from standardized scales. From this, hypotheses can be developed to be tested in pilot studies, before refining measurement tools and testing the capacity for capturing change in response to intervention.

The aims of this case study were to present an individualised case formulation for disorder-level misophonia without any co-occurring disorders, to illustrate how formulation-driven CBT for misophonia can be delivered to a patient, and to report on session-by-session change in response to interventions.

## Presenting Problem

Isabel^1^ is a 24-year-old woman who self-referred to her local primary care psychology service for support with misophonia. Since childhood, Isabel had difficulty filtering out certain everyday noises. These included sounds associated with eating, such as chewing and crunching, and muffled sounds of talking and the television in adjacent rooms. She reported feelings of irritation, anxiety and then anger in response to sounds. She would typically respond by wearing headphones, leaving the room, and waiting for others to finish eating before joining communal spaces.

Isabel told us that she did not want to appear visibly upset to others and she was worried about verbally expressing her irritation. She was aware that others did not respond in a similar way to such sounds, which contributed to feeling guilty about her reactions, frustrated, and isolated from others. She was concerned that others would judge her negatively if they were aware of her sensitivity to sounds and therefore did not ask others to change their behaviour. This caused disruption to her life, particularly at work when she had to leave the office. Isabel reported that her symptoms were getting progressively worse, resulting in her being short tempered with her closest friends, partner and family. She had not attended previous therapy for these problems.

Isabel met the criteria for misophonia, based on the revised diagnostic criteria proposed by Jager, et al. (2020a). This included preoccupation with an auditory cue, including oral sounds, intense feelings of anger and a sense of loss of control, avoidance and interference with day-to-day life, not better explained by another disorder. She scored 101 on the S-Five tool for misophonia, which was above the cut-off for significantly burdensome symptoms (Vitoratou et al., 2023). She reported no other significant psychological problems.

The S-Five (Vitoratou et al., 2021) consists of 25 statements rated from 0–10, with a total possible score of 250. There are five factors capturing different dimensions of misophonia: sense of emotional threat (e.g. “If I cannot avoid certain sounds, I feel helpless”); internalizing appraisals (“I respect myself less because of my responses to certain sounds”); externalizing appraisals (“People should do everything they can to avoid making noises that might bother others”); outbursts (“Some sounds are so unbearable that I will shout at people to make them stop”); and impact (which captures the perceived current and future limitations on seeing people and going places, rather than a comprehensive measure of the impact of the disorder; “I do not meet friends as often as I would like to because of the noises they make”). It has good psychometric properties (Cronbach’s α =.90).

Isabel’s main goal was to learn new strategies to manage the distress provoked by noises. She wanted to be less short-tempered with family and friends, have fewer disruptions to work, and to feel less guilty about her reactions towards others.

## Case formulation

**Figure 1:**
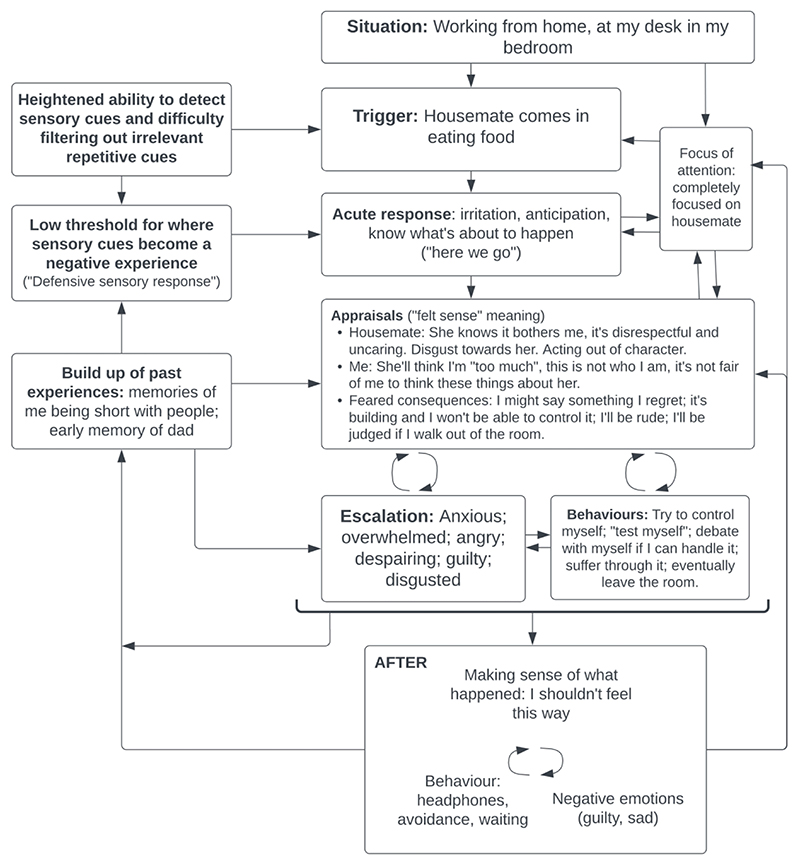
Isabel’s individualised formulation of misophonia

Using a recent example of the problem, we developed an individualised formulation (Figure 1),. Isabel reported that for as long as she could remember, she was more bothered than other people by certain sounds, in particular eating sounds and muffled noises through walls. She recalled several times as a child when she’d been short-tempered with people when she was annoyed by sounds. In the moments she was bothered by sounds, it felt like other people were being disrespectful and uncaring towards her, whilst also being aware that this was an unfair evaluation and feeling bad about judging others. She felt this was very out of character for her and developed a belief that people would find her “too much” and would judge her for the way she reacted to sounds.

The formulation included the idea that there is natural variation in the population in terms of our capacity to filter out repetitive sounds, and that her initial response of irritation and being distracted by these sounds may not be the main source of her problem. Rather, we theorised that the problem was the “felt sense” that others were uncaring if they made these sounds and that she was unfair for judging them, as well as a fear that she would snap and say something that could cause problems in her relationships. Felt sense in this context refers to interpretations and meaning that didn’t necessarily appear as verbal thoughts in the moment, where bodily sensations are used to explore the meaning of what is happening (Butler, Fennell and Hackman, 2010; Gendlin, 1996).

This led to preoccupation with not showing that she was irritated, which paradoxically increased her emotional reaction, resulting in her leaving the situation, thus missing the opportunity to disconfirm her predictions. We identified some past experiences that contributed to these appraisals and predictions, including: initially not realising that others were less bothered by these sounds (and therefore concluding that anyone who made them was doing it with full awareness of its impact); later recognising that her reactions were disproportionate and not knowing about misophonia (contributing to the feeling that she was “too much”); moments when she was short-tempered with people she cared about when she was younger (contributing to the feeling that she might snap); and memories of her mum going out of her way to please people (contributing to an inflated perception of how easily people would judge and turn against you).

## Course of treatment

Isabel was offered a course of CBT in an intensive format, a total of 12 hours of treatment over five sessions. Assessment and treatment were completed within six weeks. Her sessions were with two clinical psychologists, which was done with the combined aims of supporting the intensive format and developing the skills of one of the therapists, who had not previously completed treatment with an individual with misophonia. All sessions were conducted online using videoconferencing due to the restrictions during the COVID-19 pandemic.

Based on Isabel’s goals and her formulation, the aims of the intervention were to normalise natural variation in responsivity to sounds (similar to normalising the presence of and variation in intrusive thoughts as part of treatment for OCD), to review her felt-sense beliefs and update them through the use of imagery rescripting and behavioural experiments, to reduce distress by creating less threatening associations with sounds through behavioural experiments, and to distinguish between safety seeking behaviours and helpful coping strategies. We did not seek to eliminate sound sensitivity, but rather to reduce distress and impairment.

## Session one (four hours): formulation, Theory ABC, imagery rescripting

### Formulation

Treatment began with collaboratively developing a psychological formulation with Isabel (Figure 1). We encouraged her to close her eyes and bring to mind a recent example of being triggered by sounds. We replayed the incident and she noticed that she was re-experiencing some of the feelings she had at the time. Using imagery to reconnect with this moment, she identified beliefs that felt true in that moment, labelled her emotions and recalled what she did to cope. We identified some potential maintenance cycles.

### Theory A/B/C

The beliefs identified in the formulation were then used to develop competing theories to best explain what keeps the current problem going. This was an adapted version of the “Theory A / Theory B” strategy used for obsessive compulsive disorder, which involves seeking and comparing evidence for two competing explanations for the individual’s current problem (Bream et al., 2017). Theory A was the “violation” theory, the idea that she suffered because others were disrespectful and uncaring. Theory B was a “social threat” theory, that she was “too much”, could snap at any moment and might ruin relationships because of this flaw in her. Theory C posed that her problem could be explained by sensory over-responsivity that had worsened due to a combination of early experiences, her beliefs about herself and others, and the unintended consequences of her attempts to suppress her reactions and to avoid sounds.

In developing Theory C, we considered the potential evolutionary benefits of natural variation in how we tune into and filter out certain sounds. Isabel identified times in her life where this ability to detect and stay tuned into certain sounds had served a functional purpose. We then examined the circumstances under which this “survival tool” could cause functional impairment. We provided psychoeducation around associative learning, the role of memory and “felt sense” cognitions in our emotional experiences, and the potential for well-intended coping strategies to reinforce and maintain our distress.

**Figure 2:**
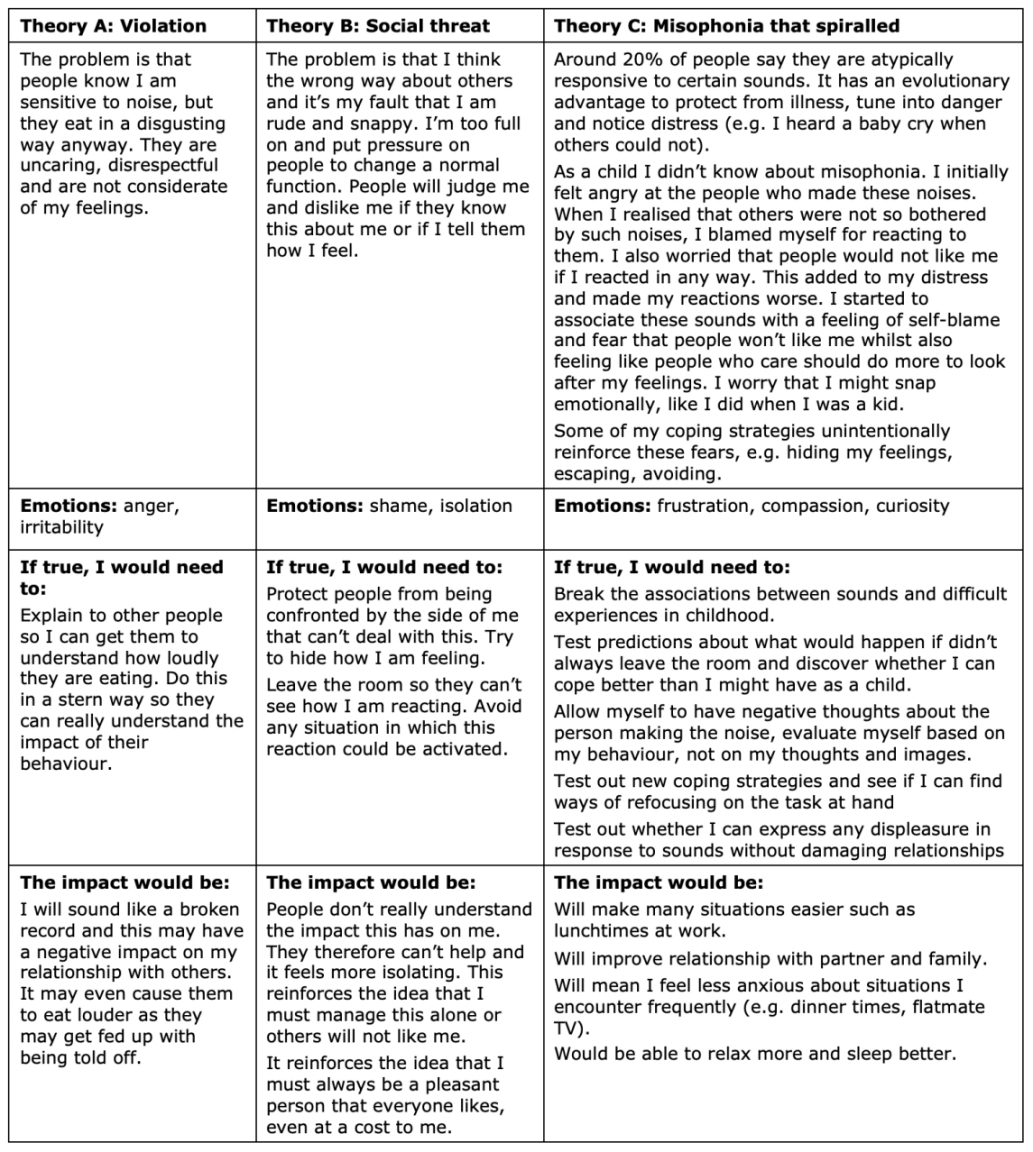
Theory A/B/C: Competing theories to explain what keeps the problem going

We then compared the three theories (Figure 2) in terms of the action required to “solve” the problem and the potential consequences of living in line with each theory. We then developed the treatment plan, with the intention of building evidence for and against each theory. Goals were developed in line with the potential outcomes from Theory C.

### Imagery Rescripting (ImRs)

The final intervention completed in the first treatment session was imagery rescripting (ImRs) of childhood memories associated with the point at which misophonia became distressing and problematic for Isabel. Imagery rescripting is an experiential technique that involves identifying and reviewing memories emotionally linked to an individual’s current difficulties. It aims to update the meaning of the experience, promoting a change in core schemas shaped by the memory (Arntz & Weertman, 1999). It is one of the core interventions used in trauma-focused CBT and schema-focused therapy and is increasingly used as part of CBT for a range of disorders (Arntz, 2012). This intervention was chosen based on the identification of early memories associated with the fear of losing control, as well as using it to see whether processing early experiences might help to update either or both of the conflicting appraisals (that it is her housemate being uncaring and that it is Isabel being unfair).

Using an “affect bridge” (for a detailed description of this technique, see van der Wijngaart, 2021) from a recent experience of being triggered by her flatmate eating pasta, Isabel identified two key memories that were emotionally linked to her current misophonic reactions: 1) her homework being disrupted by hearing muffled from her stepfather watching television; and 2) feeling distressed by eating noises from her stepfather at dinner. She reflected that while her reaction to his eating was disproportionate, it was also true that his eating was loud and messy, and that within the norms of her social and cultural background, most people would agree that it was not a polite way to eat.

Following the protocol of Arntz and Weertman (1999), Isabel was asked to bring the first memory to mind, from the perspective of herself as a child. Through discussion, she identified the unmet needs of her younger self needed: comfort and validation of her distress; to not be alone; information about why she was having such a distressing reaction to these sounds; to hear that her stepfather was not creating these sounds deliberately to upset her. The next stage involved Isabel imagining herself as an adult entering the scene, offering this care and information to her younger self. In the final stage, Isabel took the perspective of her younger self again, imagining receiving the comfort and support from her adult self.

Imagery rescripting was repeated for the second memory. Isabel updated this memory by comforting her younger self and telling her, “Your stepfather isn’t doing it on purpose to make you angry. He doesn’t realise how loud and disgusting he sounds. It is okay to feel disgusted and you can leave if it gets too much.” We then returned to the recent memory of her flatmate eating pasta in mind and introduced the same message to the recent memory. She reported that her “felt sense” belief rating of Theory A (“It’s intentional and disrespectful”) had dropped from a high to a low belief rating.

Isabel created summary statements that she wanted to carry forward from the ImRs into her day-to-day life for muffled TV sounds: “You’re not alone, it’s for a reason you react like this, you don’t need to get as stressed as you once did, it’s okay to talk to them about it (e.g. ask to turn volume down)”. For eating sounds, she wrote down: “It’s okay to feel disgusted, it’s not intentional, it’s okay to leave if it gets too much and maybe it’s okay if they know why you are leaving”.

### Between session tasks

1) Try out using the summary statements during or after moments where her emotions were high; 2) Try out asking others to make (reasonable) adjustments, for example, asking her flatmates to turn down the television; and 3) Make a note of when she has encountered difficulties putting this into practice.

## Session 2 (three hours): playing with reactions, stimulus discrimination, approaching sounds

We reviewed the previous session and Isabel reflected on between-session practice and changes during the week. She noticed that she less concerned about the sounds her flatmate had made when eating, feeling less irritation about it and taking a more reflective attitude. She said that she found the ImRs useful, noticing decreased self-judgement and the emergence of an alternative belief, “It’s okay to be disgusted by sounds.” She said that she had not previously come across the idea that it was okay to feel this way. This alternative belief was added to the formulation for further testing.

### Playing with reactions

We designed the session around exploring this new belief, as well as testing the belief that it was not fair to think judgemental thoughts about others when eating. To elicit her current beliefs about this, Isabel gave permission for one of the therapists to eat an apple in an intentionally loud way. She noticed that judgemental thoughts immediately popped into her head (“Why would anyone eat like that?!”). She said it felt like “It’s my fault that I have these thoughts” (70% belief rating) and “I shouldn’t have had those thoughts” (60% belief rating). We reviewed Theory C and came up with an alternative stance, “It’s okay to have these reactions, it doesn’t reflect anything about me as a person, it’s normal and human to have these reactions”. She gave this an 0% belief rating.

The next experiment involved “acting as if” she believed an alternative stance, using increasing exaggeration to build on her judgmental thoughts, going way beyond where her mind would usually go. She listened to the therapist eating the apple again whilst deliberately bringing up the most critical thoughts she could imagine (e.g. “No one will ever want to be in a relationship with you because you are such a disgusting pig.”). She then repeated the exercise without any deliberate action, allowing herself to experience whatever judgemental thoughts arose. We then repeated the exercise with the therapist eating a chewy sweet, again with Isabel allowing herself to experience whatever judgemental thoughts that came to mind. While this included exposure to an unpleasant sound, the aim here was exposure to her own thoughts, while not engaging in her usual behaviour of thought suppression or escape.

Isabel re-rated her beliefs throughout the exercises. By the end her initial beliefs were at 0% and the new belief (that it is okay to have these reactions) was rated at 100%. She described feeling that she had a “complete mindset shift”. She said that it had never occurred to her in the past to think that it was normal and okay to have unkind thoughts towards others, and that this did not reflect on her as a person. She concluded that her actions towards others are important but that her internal thoughts do not reflect her character, especially those thoughts that pop up automatically, that she is not choosing to have.

We then completed imagery exercises of putting this into practice in other situations. She brought to mind a range of scenarios: the muffled sounds of her flatmates from an adjacent room; her flatmate eating; her partner breathing; and her stepfather breathing. Isabel practised allowing herself to have judgmental thoughts without trying to avoid or suppress them.

### Stimulus discrimination

During these exercises Isabel noticed that she was spontaneously remembering times she had been distressed by sounds her stepfather had made whilst eating and breathing.

The concept of stimulus discrimination was introduced, and she practiced doing this in an imagery scenario of her stepdad making sounds of eating and breathing. This involved listing the similarities and differences between the past (when she didn’t know about misophonia or was blaming herself for reacting this way to his noises) and now (having been through treatment and knowing more about misophonia).

### Approaching sounds

Isabel noticed that her reactions to the sounds in the experiments earlier in the session were not as intense as she had expected. We theorised that by opting into the experiment, there was an element of control over the situation that softened the nature of her reaction. We also wondered whether her attempts to avoid and block sounds might be a safety-seeking behaviour, reinforcing the sense of threat and violation she experienced and preventing disconfirmation of her belief that she would snap or not cope. We decided to test this further with a series of experiments of “approaching” sounds rather than avoiding them.

The experiment started with one of the therapists creating a muffled TV sound by playing a TV show on an iPad covered with a blanket while Isabel tried to read. In the control condition, she tried her usual strategies of trying to ignore the sounds, then trying to block them with her fingers. In the approach condition, she tried to move towards the sound, but found that it didn’t make much difference, as we were on video call and moving closer didn’t feel like she was actually any closer to the sound. Next, we tried verbally approaching the sounds, by saying to herself “I hope they turn the volume up, come on, keep the TV on”. Then she tried returning to reading. She said that she could not focus completely, but she did notice that the intensity of her reaction went down, and she could focus a little better as compared to the control condition (attempting to ignore the sound or block it out).

### Between session tasks

1) deliberately generate judgemental thoughts (not out loud) towards others who are making sounds which she finds distressing; 2) speak to her stepfather via video call so she can practice this technique with him; 3) use stimulus discrimination if old memories surface; and 4) try out the approach response at home, inviting the muffled sounds in when hearing them and observe whether the reduced intensity of reaction that happened in the session could be replicated when she is not actually in control of the sounds at home.

## Session 3 (two hours): interacting with sounds

Session three began with reflecting on key learning points from the first two sessions and feedback from the between-session tasks. Isabel said that the most helpful intervention was the one allowing herself to have judgemental thoughts when encountering triggering sounds. She reported feeling less stress in response to the sounds and less guilt about her. An additional thing she had done between sessions was to tell her family that she was having treatment for misophonia, describing her formulation and explaining the treatment plan to them.

### Interacting with sounds (paired imagery)

The rest of the session was focused on a series of experiments of interacting with sounds in novel ways, building on the “approaching sounds” experiments from the previous session. The experiments were based on expectancy violation strategies (Frank & McKay, 2019), using imagery to change the sense of perceived control over reactions, test feared predictions and make new associations with sounds to compete with previous responses.

Isabel started by listing the sounds she had found most difficult during the week (loud eating noises with the mouth open, heavy breathing, chewing gum and muffled sounds from the television). We then generated the sounds (either pre-recorded video clips publicly available, or with one of the therapists making the sound), labelled the emotion elicited and rated the intensity. For example, the sound of eating generated a feeling of disgust at an 8 out of 10 intensity. The sound of heavy breathing elicited anxiety at an intensity of 7 out of 10.

She then identified potential alternative, non-threatening or funny sources that could be responsible for each of the sounds. For example, when hearing heavy breathing Isabel said she could think of her infant cousin breathing. For eating sounds, she thought of a cute puppy eating with its mouth open. She then practised pairing the novel imagery with the related sound, rerating the intensity of her emotional response between each trial. There was a reduction in intensity ratings for emotions for most sounds. However, her response to hearing the chewing gum sound did not change when paired with an image of a cow chewing grass, with a sustained feeling of disgusted rated at 9 out of 10. She then added in the thought “I could leave at any time if this became too much for me”, after which the intensity of the emotion reduced to 7.5 out of 10.

We agreed that it was important to continuing trying these experiments in a range of settings with different sounds and images. We discussed the advantages and disadvantages of telling her flatmates that she would be trying these exercises at home. She thought it might help test her belief that it is not acceptable to tell someone that a sound they are making is bothering her. On the other hand, she thought it might lead them to change their eating habits, giving her fewer opportunities to practise. She decided to tell one flatmate, and ask them to deliberately emphasise the sounds so that she would have more opportunities to practise as well as strengthening her alternative belief that others would be accepting of her distress, and could potentially introduce humour into it to help with the expectancy violation.

### Between session tasks

1) to ask one flatmate to deliberately make the distressing sound so she can practise bringing the novel imagery to mind; 2) to label and rate the intensity of her emotional response and then refocus her attention on an absorbing activity such as reading; 3) to practise this same response when encountering the sounds when they occur in her environment; and 4) to continue to allow herself to experience any negative or judgemental thoughts in response to distressing sounds.

## Session 4 (one hour): interacting with sounds

Isabel reported that she had practised the imagery exercise several times since the past session, with both intentional and incidental practice. She found that the unplanned practice in response to sounds naturally occurring in her environment was more effective than when she planned it with her flatmate. She reported that bringing a “cute” image to mind when encountering a distressing sound had led to the biggest decrease in intensity, especially when imagining her young cousin as the person making the sound.

### Interacting with sounds (paired imagery, labelling, attention shift)

We expanded on this exercise using humour, introducing multiple sounds at a time and practicing refocusing back on what she would rather be doing than focusing on the sounds. For example, with muffled TV sounds, she imagined her cousin as though he was the child from ‘Home Alone’ watching wildly age-inappropriate gangster films. This was practised in the session with the therapist chewing loudly, with Isabel noticing and labelling the sound, bringing the image of her cousin to mind and then refocusing back on reading her emails. We made a plan for how to increase opportunities to practice this and ways of involving her flatmates.

### Interacting with sounds (control over sounds)

Isabel reported continued high levels of distress in response to the sounds of chewing gum. She noted that not knowing when the chewing would end was causing her to feel not in control, exacerbating the distress. To experiment with increasing her perceived control, we tried using an imaginary remote control which could change the volume and speed of the sound. She would tell the therapist which button on the remote control she was pressing, and the therapist responded by changing her eating as though the remote did in fact work. Isabel reported finding this funny and said that the task distracted her from the sound itself. We then repeated the exercise, this time when she pressed the buttons the therapist didn’t actually change her behaviour, but Isabel was instructed to imagine she was in control. Next, we designed an experiment where she controlled the sounds by being the conductor in a mouth-sound orchestra. She decided to try this on a video conference call with her family making different sounds.

### Coping strategies and safety behaviours

In preparation for the final session, we discussed the various coping strategies and adjustments Isabel made to be able to function in a noisy world. We talked about giving herself permission to use strategies to cope with sounds, especially when it enabled her to do things that were important to her. We discussed the difference between healthy coping strategies and safety-seeking behaviours (i.e. strategies where the gain was outweighed by unintended negative consequences, including missing out on things and reinforcing unhelpful beliefs about her capacity to cope).

### Between-session tasks

1) continue the imagery exercise with a wide range of sounds, imagining her cousin as an alternative source of the sound; 2) practise refocusing on the task at hand once she had generated the image of her cousin making the sound; 3) practise changing the qualities of the sounds of her partner’s chewing by using an imaginary remote control; and 4) arrange a video call with her family and imagine being a conductor of their orchestra of sounds.

## Session 5 (two hours): therapy blueprint

The final session involved reviewing all of the strategies tried during therapy, identifying key learning points, discussing difficulties encountered and creating a therapy “blueprint” for continuing her progress. One of the biggest challenges was that due to the restrictions of the COVID-19 pandemic, she had been unable to see her family in person. This had meant that she had been unable to practise these strategies in person with her stepfather, whose sounds had been the original source of distress for her. She had discussed her therapy with him via video call and was planning to visit as soon as the pandemic restrictions were lifted.

Isabel’s therapy blueprint included a plan to continue practicing a range of these strategies several times a day over the coming weeks. She felt that there was a mindset change in response to these interventions, and planned to continue that shift, as well as using some of the strategies as ways of countering any potential future trigger sounds, and as a “reset” if she found herself particularly stuck with a sound.

## Outcomes

Isabel attended all sessions and was a willing and active participant in all exercises, indicating a high level of acceptability for these interventions.

**Figure 3:**
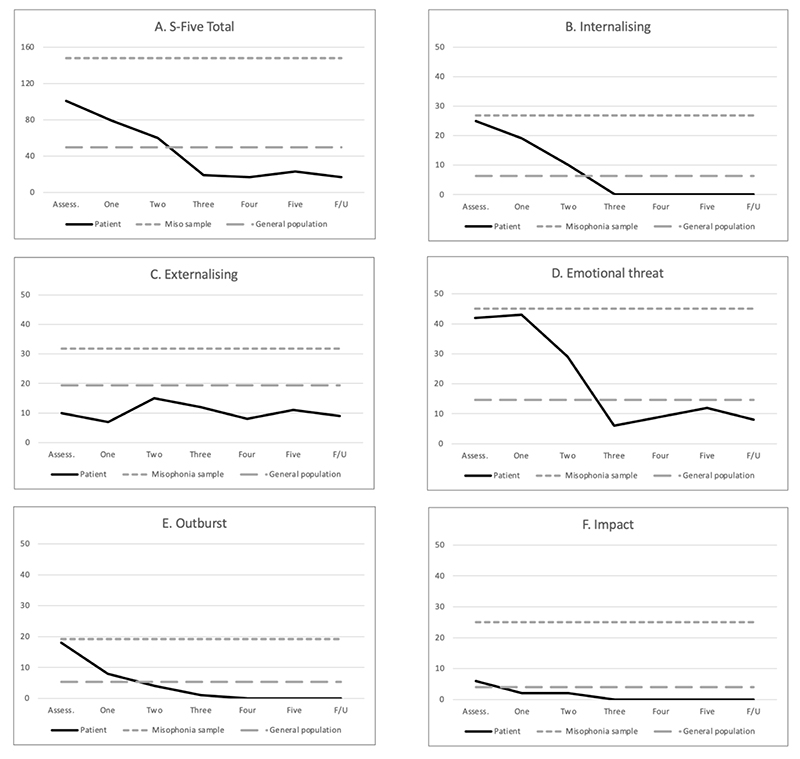
Session-by-session change on S-Five

Figure 3 shows the session-by-session change for the S-Five total and its factors, in relation to norms in a sample of individuals self-identifying as having misophonia, recruited from support groups on social media (Vitoratou et al., 2021) and in a sample representative of the UK population (Vitoratou et al., 2023). Baseline scores were close to the average scores for the misophonia sample for the factors Internalising (Figure 3B), Emotional threat (Figure 3D) and Outburst (Figure 3E), and moved to below the average score of the general population after session three, remaining below this line at follow up. For Internalizing and Outburst, the graphs show a relatively steady decrease from assessment to session three, whereas for the Emotional threat factor, the line indicates no change between assessment and session one, followed by a sharp decline between sessions one and three.

For the subscales Externalising (Figure 3C) and Impact (Figure 3F), Isabel’s scores started and remained near or below the average scores for the general population sample, indicating that these factors were not relevant parts of her clinical problem.

The Leeds Reliable Change Indicator (Morley & Dowzer, 2014) was used to calculate reliable change and clinically significant change (Jacobson & Truax, 1991) on the S-Five total score from baseline to follow up. We selected criterion c for clinically significant change, which preferred when there is an overlap in the scores between the clinical and comparison groups. This criterion states that change is clinically significant when the individual moves to closer to the mean of the comparison group than to the mean of the clinical group by the end of therapy (Jacobson & Truax, 1991). The patient’s score on the S-Five total reduced from 101 to 17, indicating both reliable and clinically significant change from baseline to follow up (RCI value = 4.29).

Visual inspection of the graph (Figure 3A) showed a sharper drop between sessions 2 and 3. To examine this further, we used the reliable change indicator to test for reliable change between each session. That is, we entered the session 1 score as pre-treatment and the session 2 as post-treatment, to determine whether the changes made between sessions 1 and 2 could be considered reliable change, then repeated this for each session. The decrease in scores between sessions 2 and 3 was the only between-session score that was considered reliable and clinically significant change (RCI value = 2.09).

Table 2 shows the results of the S-Five Trigger supplementary scale (S-Five-T), which measures the nature and intensity of reaction to 37 trigger sounds. We have reported on all trigger items for which there was an initial score of 8 or above in intensity. For all trigger items not reported here (i.e. those that were rated as 7 and below at baseline), there was a score of 0 or 1 intensity at follow up. The sounds “chewing gum loudly” and “chewing loudly” reduced from a 10 to an 8 and 7, respectively, at follow up. All other sounds reduced to 5 and below. “Chewing gum loudly” was rated as causing a distress reaction at follow up, all other sounds were rated as causing irritation or no feeling. For all sounds except chewing gum, the nature of the reaction changed to irritation or no feeling by the start of the third session.

## Discussion

This case study aimed to demonstrate how formulation-driven CBT can be delivered to a patient with misophonia. We provided detailed descriptions of how interventions were selected and delivered, adapting the treatment plan iteratively as new information emerged.

The outcome data showed good support for progress on Isabel’s goals. She wanted to learn new strategies to manage the distress provoked by noises. By the end of treatment, only one trigger sound caused a feeling of distress (chewing gum) and she had adopted two strategies for use in those moments: visualising the sound coming from another source (e.g. her cousin making the sound) and pretending she was in control of the sound, by using an imaginary remote control. Additionally, the Emotional threat factor reduced to lower than the average score for a community sample, indicating that she no longer experienced feeling trapped, distressed and helpless in relation to sounds.

Another goal was to be less short-tempered with family and friends, which is demonstrated by scores on the Outburst factor reducing to zero, and the S-Five-T showing that anger was no longer a primary response to any of her trigger sounds. Her goal to feel less guilty about her reactions was measured by her scores on the Internalising factor reducing to zero. Her final goal was around disruptions to work, which was difficult to measure as treatment took place while she was working from home during the COVID-19 pandemic. She planned to try to generalise this learning to the work environment and in-person family events when government regulations allowed for this.

Another aim of this case study was to examine the session-by-session change for this patient. Visual inspection of the S-Five graphs shows an incremental improvement in both Internalising and Outbursts from the assessment up to the beginning session three, after which the improvements were maintained through to follow up. There are no clear points of “sudden gain” for these two factors, so it is difficult to tell if there were any specific interventions that contributed to these changes. It is possible that this change could have happened regardless of the nature of the intervention, that it was the process of being in supportive therapy itself that contributed to this change. With the Emotional threat factor, the graph shows a straight line from assessment to session one, and then a rapid drop between sessions one and three. This suggests that active intervention phase was likely to have contributed to this change, although again it is not clear whether it is the result of these specific interventions, or whether any CBT intervention would have seen a similar change. This could be investigated further using single case experimental design with a rebaseline phase between specific interventions.

The session-by-session analysis revealed that there was reliable change in the overall severity of misophonia (S-Five total score) between sessions two and three, but not between any other sessions. Without an experimental design, it is hard to interpret these results. It is possible that the content of session two, in particular, was more clinically meaningful for this patient. It’s also possible that this was a cumulative effect, building over sessions rather than attributable to the content of that session. Looking closer at the content of session two, it was the first session where Isabel encountered sounds as part of her experiments. Using the inhibitory learning framework for misophonia described by Frank and McKay (2019), the improvements could be explained by having the patient’s expectations violated (e.g. she did not “snap” when she encountered sounds), which is supported by her feedback during the session that she coped better than she expected.

However, there was also symptom improvement occurring before starting the exercises involving the use of sounds, indicating that sound-based strategies were not the only contributing factor to change. Isabel reported that she found the imagery rescripting (ImRs) helpful. Her belief rating for Theory A (“It’s intentional and disrespectful”) changed from high to low following this intervention. She also noticed that it led to reduced self-judgement and it helped with generating alternative beliefs that she had not previously considered. She said that the “complete mindset change” happened after doing experiments where she listened to sounds and exaggerated her responses without judging those thoughts, suggesting that it was useful to have the combination of ImRs and playing with reactions in response to sounds.

It was interesting to note that while her overall severity scores decreased, she still reported that many sounds would cause an irritation response. This outcome fits with the finding that misophonia severity was negatively correlated to the frequency with which someone reports irritation as a response to sounds, suggesting that irritation in response to sounds is not indicative of the latent trait of misophonia the “disorder” (Vitoratou et al., 2021). In a sample representative of the UK population, only 15% of people reported no feeling in response to loud chewing, with most people reporting either irritation or disgust. Twenty-four percent of people reported irritation in response to clock ticking, 45% to muffled sounds and 32% to normal volume eating sounds (Vitoratou et al., 2023).

The outcomes suggest that CBT was helpful for reducing the severity of misophonia in this patient. Her reduction in scores on the S-Five were maintained through to one month follow up, and the intensity rating for some triggers sounds reduced further between her final session and follow up. This was further supported by her feedback that she had experienced a “complete mindset change”. Whilst we are limited by not having a longer follow up period, the results are promising.

This detailed case study also provides some preliminary information about possible change characteristics for misophonia, and initial proof of concept for the S-Five’s capacity to capture meaningful change in this patient group. In particular, we see evidence that areas of change for this patient group could include a reduced sense of feeling trapped and helpless, as captured by the Emotional Threat factor of the S-Five, self-blame and self-judgement captured by the Internalising factor, and aggression (or fear of aggression), captured by the Outbursts factor.

Additionally, Isabel reported a change in the felt-sense belief that others’ behaviour was intentional and disrespectful. It was interesting to note that this belief was not reflected in the Externalising factor, which purports to measure the attribution of blame onto others (Vitoratou et al., 2021). The relative difference between the clinical and community norms (around 50% lower in the community sample) for this factor is much smaller compared to the other S-Five factors (3-4 times lower in the community sample than the misophonia sample) (Vitoratou et al., 2023), which could mean that the externalising factor is possibly not a key feature in distinguishing those with and without misophonia, or that the current items in this factor do not adequately capture this concept. Further examination of this would be useful, particularly in relation to what might best capture change in response to intervention.

The intensive format of this treatment was both a strength and a limitation. The longer sessions enabled us to try multiple variations of experiments and to build on and quickly test ideas that emerged during the session. From an inhibitory learning perspective, this promoted “deepened extinction” by combining multiple stimulus cues and generalisability through variability in the way we delivered the experiments (Craske et al., 2014). However, by completing multiple interventions in each session, we are unable to make strong hypotheses about which interventions most contributed to change. Further studies using single case experimental design are needed.

One further area of research needed is examining the difference between adaptive coping strategies and safety seeking behaviours in misophonia. Frank and McKay (2019) hypothesised that the unintended consequences of misophonic reactions may reinforce the meaning applied to the sound, thus intensifying reactions. This is in line with maintenance cycles for a range of disorders. However, in our clinical experience, patients have told us that certain coping strategies are vital for being able to function, for example using headphones to concentrate on work or study, or using music to improve their experience of mealtimes. Further research on this topic is needed.

We recommend that therapists working with this patient group use functional analysis and behavioural experiments to test the intended and unintended consequences of the individual’s strategies. We advise therapists and patients that some strategies may even serve as an adaptive coping strategy in certain situations (e.g. using earplugs for restful sleep) and a safety behaviour in other circumstance (e.g. wearing earplugs all day, possibly reinforcing the belief that one cannot cope if they encounter sounds, and unintentionally increasing their auditory sensitivity as a result of blocking sounds all day).

In conclusion, a formulation-driven CBT approach appeared to be useful for Isabel. The combined outcomes of symptom reduction, belief shifts and progress towards goals indicates that there were cognitive and behavioural factors contributing to her distress and impairment. This adds to the building evidence suggesting that it is worth pursuing the development of a theoretical cognitive model of distress in misophonia. To identify themes in potential mechanisms of change for this disorder, research should include single case experimental design using multidimensional measurement tools and in-depth qualitative exploration of the experience of having misophonia.

## Figures and Tables

**Table 1:** Overview of session content

Session	Duration	Content
Assessment	2 hours	Established primary problem and assessed suitability for CBT
One	4 hours	Case formulation, Theory ABC, imagery rescripting
Two	3 hours	Playing with reactions, stimulus discrimination, approaching sounds
Three	2 hours	Interacting with sounds (paired imagery exercises)
Four	1 hour	Interacting with sounds (paired imagery, labelling sounds, shifting attention and using control over sounds), distinguishing between coping strategies and safety seeking behaviours
Five	2 hours	Therapy blueprint
Follow up	No session	One month follow up outcome measures collected by email

**Table 2:** Outcomes for the S-Five trigger scale

Triggers	Assess.	Session One	Session Two	Session Three	Session Four	Session Five	1m Follow Up
S-Five-T: Intensity of reaction							
Normal eating	9	9	6	6	5	8	3
Lip smacking	8	8	6	5	0	0	1
Chewing gum loudly	10	9	8	9	9	9	8
Slurping	8	6	5	0	0	0	2
Loud chewing	10	10	8	7	8	9	7
Teeth sucking	8	5	4	0	0	0	0
Crunching eating	9	8	7	5	7	7	5
Normal breathing	8	3	3	0	0	2	0
Loud breathing	9	8	7	7	5	8	3
Muffled sounds	9	8	7	7	5	7	4
Clock ticking	8	5	3	3	3	4	3
S-Five-T: Nature of reaction							
Normal eating	Distress	Distress	Irritation	Irritation	Irritation	Irritation	Irritation
Lip smacking	Disgust	Distress	Irritation	Irritation	No feeling	No feeling	Irritation
Chewing gum loudly	Anger	Disgust	Disgust	Disgust	Disgust	Disgust	Distress
Slurping	Irritation	Irritation	Irritation	No feeling	No feeling	No feeling	Irritation
Loud chewing	Anger	Anger	Disgust	Irritation	Irritation	Disgust	Irritation
Crunching eating	Distress	Irritation	Irritation	Irritation	Irritation	Irritation	Irritation
Teeth sucking	Irritation	Irritation	Irritation	No feeling	No feeling	No feeling	No feeling
Normal breathing	Irritation	Irritation	Irritation	No feeling	No feeling	No feeling	No feeling
Loud breathing	Anger	Distress	Distress	Irritation	Irritation	Irritation	Irritation
Muffled sounds	Distress	Distress	Irritation	Irritation	Irritation	Irritation	Irritation
Clock ticking	Irritation	Irritation	Irritation	Irritation	Irritation	Irritation	Irritation

Note: Questionnaires were completed *before* each session Reactions have been shaded to visualise change, with darker shading indicating more misophonia-specific emotions and high intensity (8-10), lighter shading to show irritation, which can be experienced by those with and without misophonia and moderate intensity (5-7) and no shading for low intensity (0-4) and “no feeling”. Session 1. Formulation, Theory ABC, ImRs Session 2. Reactions, stimulus discrimination, approaching Session 3. Interacting with sounds (paired imagery) Session 4. Interacting with sounds (paired imagery + control) Session 5. Blueprint Follow up was done by email and did not include a session.

## Data Availability

The data that support the findings of this study are available on request from the corresponding author. The data are not publicly available as they contain information that could compromise the privacy of the subject.

## Key Learning Aims

- To understand misophonic distress from a CBT perspective
- To learn a formulation-driven approach to misophonia
- To apply transdiagnostic interventions to misophonia
- To learn about misophonia-specific interventions
- To consider the value of a multi-dimensional measure of misophonia

## Key Practice Points

- Treatment for misophonia should be formulation-driven
- Novel engagement with sounds may be a key part of treatment
- ImRs can support processing of memories in misophonia
- The S-Five shows promise at capturing change in misophonia

## Footnotes

1 We have used a pseudonym and changed some details to protect the identity of the patient. Informed consent was given by the patient, who read the paper and agreed that the details were sufficiently changed.
